# Metabolic reprogramming in diabetic panvascular disease: Molecular mechanism and therapeutic strategies

**DOI:** 10.1016/j.isci.2026.117578

**Published:** 2026-09-18

**Authors:** Yajing Bai, Yinghong Bai, Shiwei Liu

**Affiliations:** 1 Third Hospital of Shanxi Medical University, Shanxi Bethune Hospital, Shanxi Academy of Medical Sciences, Tongji Shanxi Hospital, Taiyuan 030032, China; 2 Department of Endocrinology, Third Hospital of Shanxi Medical University, Shanxi Bethune Hospital, Shanxi Academy of Medical Sciences, Tongji Shanxi Hospital, Taiyuan 030032, China; 3 Department of Neurosurgery, Jinzhong Hospital of Shanxi Medical University, No. 689, Huitong South Road, Yuci District, Jinzhong City 030600, Shanxi Province, China

**Keywords:** diabetic panvascular disease, metabolic reprogramming, diabetic complications

## Abstract

Diabetes mellitus substantially increases the risk of cardiovascular, cerebrovascular, renal, retinal and peripheral vascular complications. These are collectively referred to as diabetic panvascular disease. A traditional approach that treats each complication separately is no longer sufficient. An emerging framework places metabolic reprogramming at its center. This review examines how the diabetic environment, including hyperglycemia, insulin resistance, and dyslipidemia, rewires core metabolic pathways in vascular cells. Major changes include the diversion of glucose through the polyol, hexosamine and AGE-PKC axes, lipotoxicity resulting from excessive fatty acids, disruption of amino acid networks such as BCAA and one-carbon metabolism, mitochondrial dysfunction with increased mtROS production, and epigenetic modifications that drive metabolic memory. These interconnected abnormalities promote oxidative stress and chronic inflammation, which in turn lead to endothelial dysfunction, atherosclerosis, microvascular rarefaction, and end-organ damage in the heart, brain, kidney, retina, and peripheral nerves. We also describe the current therapeutic landscape, ranging from established agents such as metformin, SGLT2 inhibitors, GLP-1 receptor agonists, RAAS blockers, and anti-VEGF therapies to emerging mechanism-based strategies including aldose reductase inhibitors, glucokinase activators, PKC, RAGE, NOX, and NLRP3 inhibitors, miRNA modulators, and exosome therapy. Finally, we highlight unmet needs and future directions. These may include multi-omics guided precision phenotyping, cross-organ causal inference, single-cell metabolic flux technologies, and adaptive clinical trial designs. By shifting from a glucose-centric to a metabolic reprogramming-centric paradigm, this review aims to provide a roadmap for developing more targeted therapies for diabetic panvascular disease.

## Graphical abstract

**Figure:**
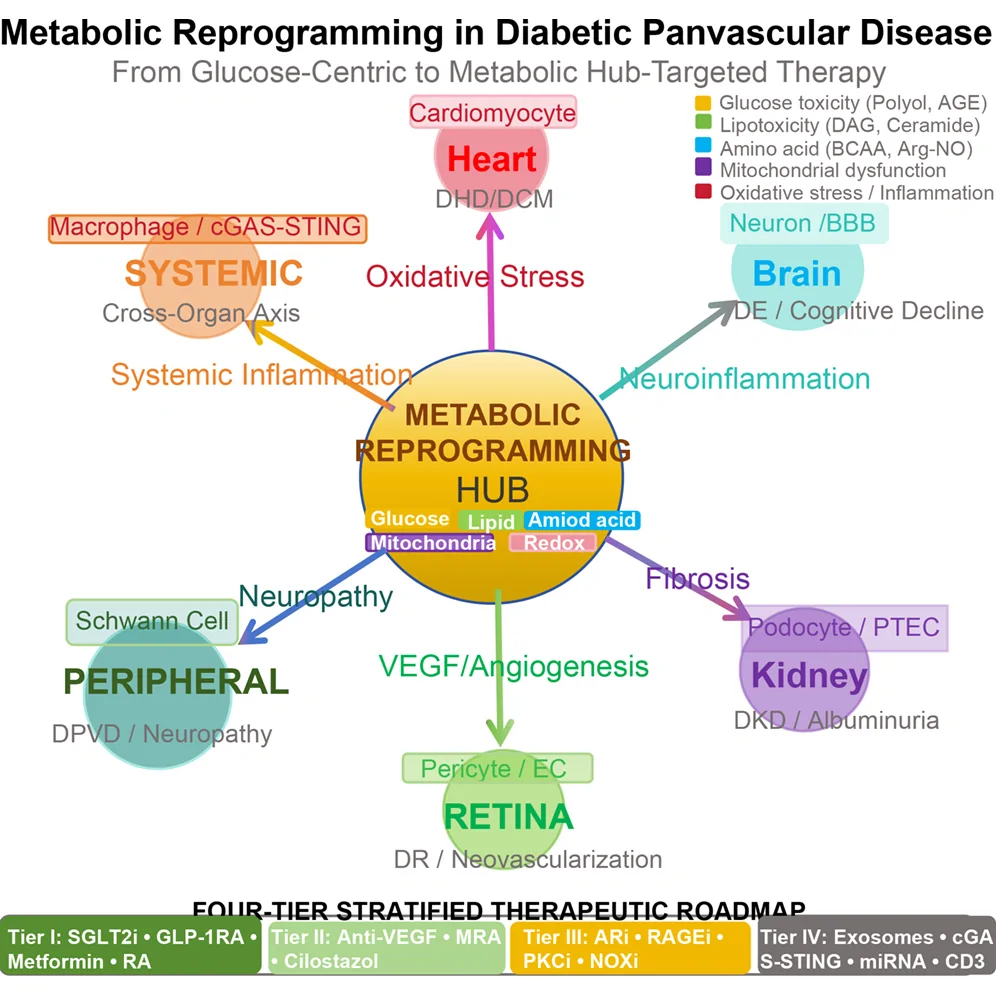

## Teaser

Health sciences

## Introduction

Diabetes mellitus, particularly type 2 diabetes, poses a global public health crisis. In 2010, the global prevalence of diabetes in adults was 6.4%, affecting 285 million adults, and is projected to increase to 7.7% by 2030, reaching 439 million.^1^ This epidemic is inextricably linked to a surge in diabetic complications, which are the primary drivers of diabetes-related morbidity, mortality, and healthcare costs.^1^ Cardiovascular disease remains the leading cause of death in patients with diabetes, who face a 2- to 4-fold increased risk compared to their non-diabetic counterparts.^1^ Beyond traditional macrovascular outcomes such as myocardial infarction and stroke, diabetes triggers a constellation of microvascular complications—including nephropathy, retinopathy, and neuropathy—that severely impair quality of life.^1^^,^^2^

The traditional clinical approach, which often treats these complications in isolation, is no longer adequate for comprehensive long-term management of patients with diabetes. A paradigm shift toward a more integrated view has led to the concept of diabetic panvascular disease (DPD).^3^ DPD is defined as a clinical syndrome in which vessels of all sizes—from large arteries to microvessels in the heart, brain, kidney, retina, and peripheral systems—are affected by a common pathological process, with atherosclerosis as its core feature.^3^

The unifying framework of DPD is not merely a semantic consolidation of existing knowledge; it proposes a specific mechanistic logic, namely that diverse vascular complications share a common pathogenic soil of systemic metabolic reprogramming and evolve through dynamically interacting rather than independent pathways. This framework distinguishes itself from previous reviews, which typically cataloged individual metabolic abnormalities (such as the polyol pathway or AGE-RAGE axis) within single organ systems, by emphasizing the interconnected and network-driven nature of the disorder across multiple vascular territories. Within this framework, DPD is conceived as a multi-node, network-driven disorder, with metabolic reprogramming serving as the central hub that connects glucose, lipid, and amino acid dysregulation to oxidative stress, inflammation, and epigenetic modifications, ultimately propagating injury across the heart, brain, kidney, retina, and peripheral vessels. The novelty of this perspective lies not in identifying each pathway for the first time, but in systematically integrating them into a cross-organ network and proposing that therapeutic targeting of shared metabolic hubs may confer simultaneous protection across multiple vascular beds.

Clinical reality involves overlapping pathologies: macrovascular atherosclerosis often coexists with microvascular dysfunction, basement membrane thickening, and microthrombosis. For example, while diabetic kidney disease and retinopathy are predominantly microangiopathic, the heart and brain suffer from a damaging combination of both^3^ (Figure 1).

**Figure 1:**
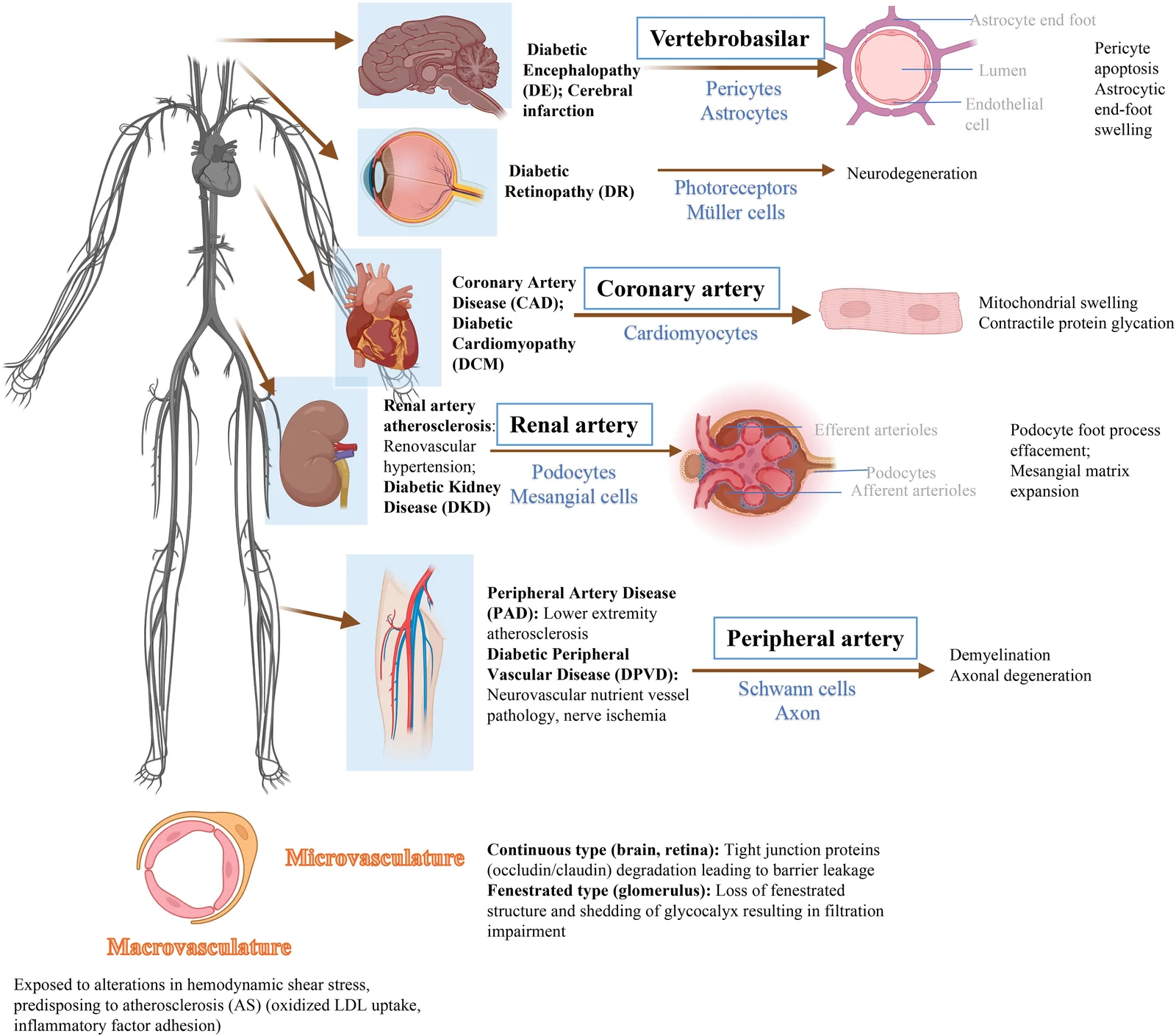
Overview of organs and vascular beds involved in diabetic panvascular disease This figure summarises the major macrovascular and microvascular beds affected by DPD, including the vertebrobasilar artery/brain, coronary artery/heart, renal artery/glomerulus, and peripheral arteries/neurovascular unit. Key cell types and representative pathological changes are outlined. Continuous microvessels (brain, retina) are characterised by tight junction degradation and barrier leakage, whereas fenestrated microvessels (glomerulus) show loss of fenestrated structure and glycocalyx shedding, leading to filtration impairment.

This complex network of vascular damage is not merely a collection of independent events but is driven by shared, fundamental molecular mechanisms. Among these, metabolic reprogramming has emerged as a central, unifying theme. The diabetic milieu—characterized by hyperglycemia, insulin resistance, and dyslipidemia—forces a pathological rewiring of metabolic pathways in vascular cells. This includes a toxic diversion of glucose through the polyol and hexosamine pathways, an over-reliance on fatty acid oxidation leading to lipotoxicity, disrupted amino acid metabolism, and profound mitochondrial dysfunction. These metabolic disturbances, in turn, fuel a vicious cycle of oxidative stress, chronic inflammation, and epigenetic changes that perpetuate vascular injury across all target organs. Understanding this metabolic reprogramming is not only an academic exercise; it is the key to unlocking novel therapeutic strategies that go beyond simple glycemic control to directly target the root causes of DPD progression. This review will systematically examine the organ-specific manifestations of DPD—diabetic heart disease (DHD), diabetic encephalopathy (DE), diabetic kidney disease (DKD), diabetic retinopathy (DR), and diabetic peripheral vascular disease (DPVD)—through this unifying metabolic framework.

This article is presented as a narrative review intended to synthesize heterogeneous observations across preclinical and clinical studies and generate testable hypotheses, rather than to assert a definitive causal model. Literature was identified through searches of PubMed, Embase, and Web of Science using the terms “diabetic panvascular disease,” “metabolic reprogramming,” “diabetic complications,” “mitochondrial dysfunction,” “oxidative stress,” “endothelial dysfunction,” “lipotoxicity,” “advanced glycation end-products,” “SGLT2 inhibitor,” and “GLP-1 receptor agonist,” with no language restriction and covering publications up to August 2026. We use a metabolic reprogramming-centric framework organized at pathway, cell, and organ levels to structure the literature and enhance clarity in interpreting the evidence landscape. Current evidence is largely derived from mechanistic studies in rodent models, *in vitro* cell cultures, and retrospective clinical cohorts with variable metabolic phenotyping, inconsistent biomarker assays, and heterogeneous definitions of diabetic panvascular disease across target organs, which limits direct comparability across studies. The metabolic reprogramming framework is therefore used as a heuristic to connect glucose, lipid, and amino acid metabolism disturbances with mitochondrial dysfunction, epigenetic modifications, and end-organ vascular injury as reported in different experimental and clinical designs, while explicitly maintaining uncertainty where directionality, temporality, tissue specificity, and generalizability to human disease remain unproven.

## Overview of metabolic reprogramming pathways in DPD

The diverse vascular complications of diabetes are unified by common pathways of metabolic dysregulation. The diabetic state reprograms cellular metabolism, creating a self-perpetuating cycle of oxidative stress, inflammation, and vascular damage. As shown in Figure 2, under diabetic conditions, endothelial cells, smooth muscle cells, cardiomyocytes, intrinsic glomerular cells, neurons, and immune cells collectively converge on five core metabolic abnormalities: glucose metabolism disorder, lipid metabolism abnormality, amino acid metabolism disorder, mitochondrial dysfunction, and redox homeostasis imbalance. Downstream, these abnormalities connect to modules including epigenetic regulation, inflammatory signaling, the oxidative stress core node, glycometabolic enzymes, hormone signaling, vasoactive factors, and lipid mediators, ultimately driving end-organ phenotypes such as myocardial infarction, cerebral infarction, and diabetic retinopathy. This framework suggests that DPD is not a single-pathway disease but a multi-node, network-driven disorder.

**Figure 2:**
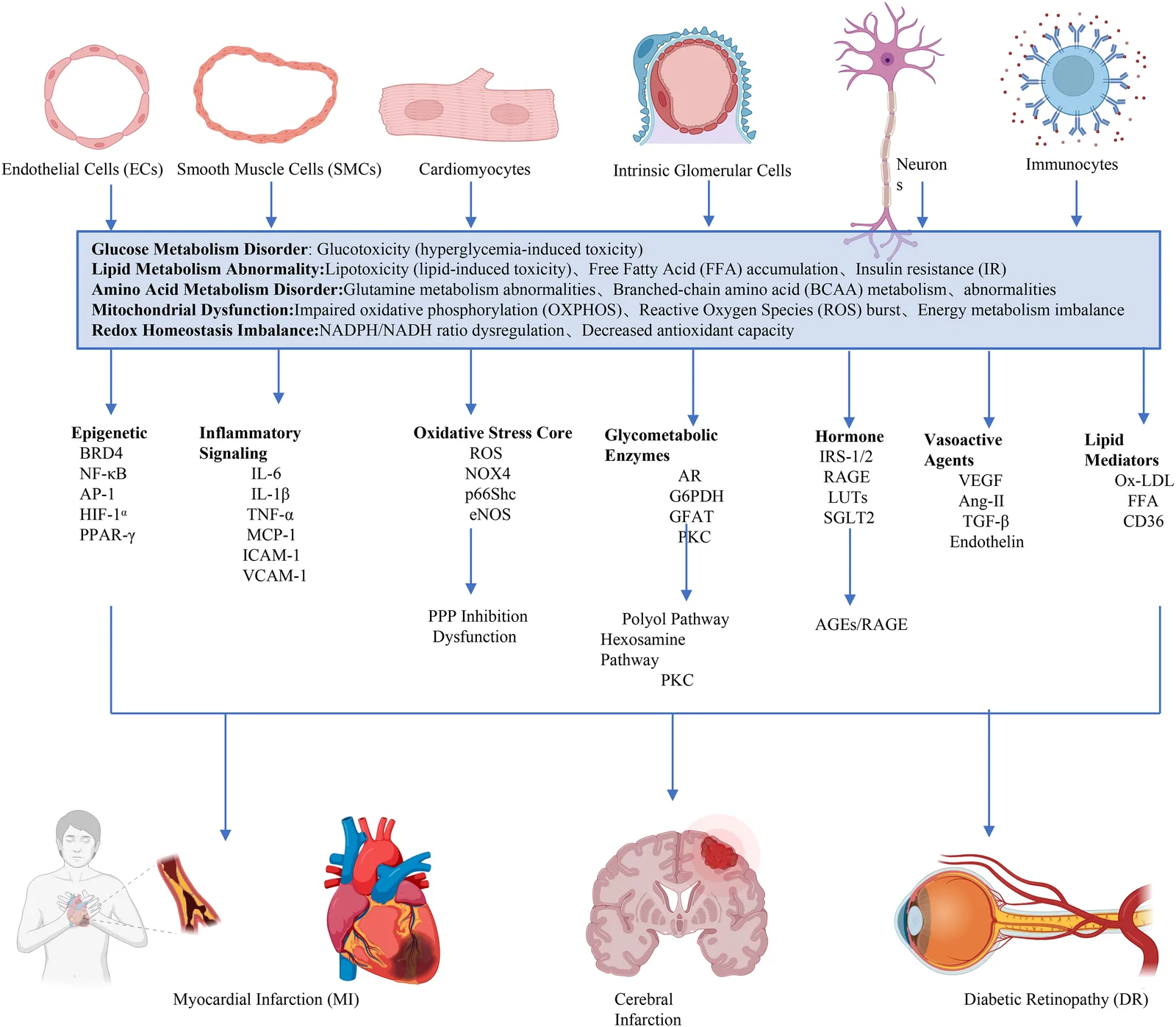
Overview of the metabolic reprogramming and signaling network in diabetic panvascular disease (DPD) This figure summarizes the mechanistic network of DPD across four levels: cell types, metabolic abnormalities, key signaling nodes, and organ outcomes. Upstream components encompass abnormalities in glucose, lipid, and amino acid metabolism, mitochondrial dysfunction, and redox imbalance. Midstream components include epigenetic/transcriptional regulators (such as BRD4, NF-κB, AP-1, HIF-1α, and PPAR-γ) and key effector molecules (including IL-6, IL-1β, TNF-α, NOX4, p66Shc, eNOS, AR, GFAT, PKC, RAGE, VEGF, Ang II, TGF-β, and CD36). Downstream consequences ultimately manifest as pathological phenotypes, including microcirculatory disturbances and parenchymal cell damage in target organs such as the heart, brain, retina, and peripheral vasculature.

### Glucose metabolic redirection: The glycotoxicity cascade

Chronic hyperglycemia is the primary initiator of diabetic vascular injury. High glucose forces glucose flux through alternative metabolic routes, most notably the polyol pathway. There, aldose reductase converts glucose to sorbitol, consuming NADPH. This NADPH depletion reduces the regeneration of the critical antioxidant glutathione, inducing oxidative stress.^4^^,^^5^ This pathway is involved in the development of retinopathy,^6^^,^^7^ nephropathy,^8^ and neuropathy Cortese et al.^9^ In the retina, high glucose induces pericyte apoptosis via the PKC-δ/p38α MAPK/SHP-1 pathway, leading to PDGF resistance and the formation of acellular capillaries, a hallmark of early DR.^10^ This process is linked to N6-methyladenosine (m6A) RNA modification, where METTL3-mediated m6A methylation epigenetically regulates pericyte dysfunction.^11^

A direct consequence of hyperglycemia is the non-enzymatic formation of advanced glycation end-products (AGEs).^1^ AGEs are directly toxic to cells and exert their effects mainly by binding to their receptor, RAGE. This ligation activates NADPH oxidase, generating a burst of reactive oxygen species (ROS).^4^^,^^12^ The AGE-RAGE-ROS axis is a master switch for inflammation, triggering NF-κB and leading to the expression of pro-inflammatory cytokines and adhesion molecules.^4^^,^^13^ This cascade is central to DCM,^1^ nephropathy,^14^ and vasculopathy.^1^ For example, AGEs directly bind MD2, the co-receptor of TLR4, forming an AGEs-MD2-TLR4 complex that drives inflammation in DCM.^1^ Moreover, ROS-induced PKCδ activation links AGEs to mitochondrial dysfunction and cardiomyocyte apoptosis.^1^ The AGE-RAGE axis also promotes EndMT, contributing to cardiac fibrosis.^15^ In the kidney, AGE-RAGE-induced cytosolic ROS promotes mitochondrial superoxide production, thereby exacerbating DKD.

### Lipid metabolism dysregulation and lipotoxicity

In the insulin-resistant state, the heart and other tissues become metabolically inflexible, shifting their energy preference from glucose to fatty acid oxidation (FAO).^1^^,^^16^ This leads to excessive uptake and oxidation of free fatty acids (FFAs). Elevated plasma FFA is an independent risk factor for coronary heart disease in patients with T2DM.^1^ This lipid overload results in the accumulation of toxic intermediates such as diacylglycerol (DAG) and ceramides in non-adipose tissues—a phenomenon known as lipotoxicity. DAG activates PKC isoforms, impairing insulin signaling and exacerbating insulin resistance, thereby creating a vicious cycle.^1^ In the heart, this lipid overload leads to mitochondrial dysfunction, ER stress, and apoptosis, driving ventricular remodeling and heart failure.^16^ In the kidney, lipid accumulation is observed in proximal tubules of patients with DKD, mediated by upregulation of ADRP and SREBP-1, which correlates with tubulointerstitial injury and inflammation.^17^ Similarly, in podocytes, lipid accumulation is a major determinant of injury, with JAML playing a key role by regulating lipid metabolism via the SIRT1-mediated SREBP1 pathway.^18^

Beyond intracellular accumulation, lipoproteins themselves are modified. Oxidized LDL (oxLDL) is a key player in atherogenesis. The oxLDL/LDL-C ratio is a potential biomarker for severe coronary atherosclerosis in patients with diabetes.^1^ oxLDL activates TLR4 and its co-receptor MD2, causing injury to retinal Müller cells. Fatty acid-binding proteins also play critical roles. Adipocyte FABP (A-FABP) acts as a lipidokine; it is induced in macrophages/microglia after cerebral ischemia and promotes BBB disruption by upregulating MMP-9 via the JNK/c-Jun pathway.^19^ Circulating A-FABP levels positively correlate with infarct volume in patients with stroke.^19^ CD36, a scavenger receptor for oxLDL, is involved in atherosclerosis, and its expression in monocytes is modulated by hyperglycemia.^20^

It is worth noting, however, that the pathological roles of inflammatory mediators in the context of metabolic dysregulation are highly context-dependent. A paradigm example is interleukin-6 (IL-6), which exhibits a dual role in metabolic adaptation. Under physiological conditions, such as during exercise, IL-6 functions as a beneficial myokine that promotes fatty acid oxidation and insulin sensitivity via the AMPK pathway. In contrast, under chronic diabetic conditions, persistently elevated IL-6 drives systemic insulin resistance, endothelial dysfunction, and vascular inflammation. This context-dependent “yin-yang” effect, as comprehensively reviewed by Wang et al.,^21^ underscores that simply categorizing inflammation as uniformly harmful oversimplifies the complex interplay between metabolic stress and immune adaptation.

### Amino acid network disruption

Amino acid metabolism is profoundly altered in diabetes and contributes to vascular dysfunction. Branched-chain amino acids (BCAAs) are often elevated in insulin-resistant states, and their dysregulation is linked to cardiovascular disease. A landmark study identified a variant near the *GLUL* gene that is specifically associated with coronary heart disease risk in diabetic (but not non-diabetic) individuals, implicating glutamate-ammonia ligase and the γ-glutamyl cycle in diabetes-accelerated atherosclerosis.^22^ Furthermore, impaired influx into the TCA cycle, reflected by altered levels of amino acids such as fumarate, asparagine, and glutamine, is associated with the progression of cardiovascular autonomic neuropathy in T1DM, independent of glycemic control.^23^

The L-arginine/nitric oxide (NO) axis is essential for vascular health. Endothelial NO synthase (eNOS) produces NO from L-arginine, a process critical for vasodilation and endothelial function.^24^ In diabetes, this axis is disrupted. eNOS can become “uncoupled”, producing superoxide instead of NO. Both endothelial and red blood cell eNOS independently contribute to blood pressure regulation.^25^ Strategies to enhance NO production, such as L-arginine supplementation or eNOS gene delivery, have shown promise in preclinical models of endothelial injury and hindlimb ischemia.^26^ Conversely, arginase competes with eNOS for the common substrate L-arginine. Arginase inhibition improves endothelial function in patients with hypercholesterolaemic, highlighting its potential as a therapeutic target.^27^

Serine and glycine metabolism, which fuels one-carbon metabolism via the folate cycle, is also critical. This pathway supports nucleotide synthesis, methylation reactions, and antioxidant defense.^28^^,^^29^ In activated macrophages, LPS stimulates the serine synthesis pathway and one-carbon metabolism to drive epigenetic reprogramming and IL-1β expression through the production of S-adenosylmethionine (SAM).^30^ This demonstrates a direct link between amino acid metabolism, epigenetic state, and inflammation.

### Mitochondrial imbalance: The energy-failure-inflammation axis

Mitochondria are central to energy production, but in diabetes they become dysfunctional and a major source of pathological signals. Hyperglycemia and lipotoxicity increase substrate flux through the electron transport chain (ETC), leading to excessive mitochondrial ROS (mtROS) production.^31^ This overproduction is a unifying mechanism of diabetic complications, activating pathways such as polyol flux, AGE formation, and PKC.^4^^,^^32^ Mitochondrial dysfunction is not uniform across tissues; in T1DM, heart mitochondria show reduced respiration and ATP synthesis, while kidney and brain mitochondria are preserved, underscoring the central role of mitochondrial dysfunction in DCM.^33^

Damaged mitochondria also trigger inflammation. mtROS can activate the NLRP3 inflammasome, leading to IL-1β maturation and inflammation. This mtROS-TXNIP-NLRP3/IL-1β axis contributes to tubular injury in DKD.^34^ The AGE-RAGE pathway also exacerbates mitochondrial damage; AGE-induced cytosolic ROS triggers a mitochondrial permeability transition, which, in the presence of hyperglycemia, further fuels mtROS production from complex I, creating a vicious cycle.^14^ Interestingly, ROS-induced ROS release mediated by the Nox4/Nox2/p66Shc signaling axis amplifies mitochondrial ROS and sustains VEGFR2 signaling during angiogenesis, illustrating the complex, context-dependent role of ROS.^35^

Moreover, mitochondrial dysfunction serves as a critical upstream trigger for a network of regulated cell death (RCD) modes that extend beyond classical apoptosis. As systematically elucidated by Qi et al.^36^ in the context of myocardial ischemia-reperfusion injury, metabolic stress and mitochondrial damage orchestrate a multi-modal death network encompassing apoptosis, necroptosis, ferroptosis, pyroptosis, and cuproptosis. This interconnected network, rather than a single isolated pathway, determines the ultimate fate of stressed cells and contributes to the progressive organ damage observed in diabetic complications, particularly in diabetic cardiomyopathy.

Beyond mitochondrial dysfunction, endoplasmic reticulum (ER) stress serves as a core pathological hub that bridges metabolic dysregulation, oxidative stress, and inflammation in DPD. Under diabetic conditions, persistent hyperglycemia, lipotoxicity, and elevated reactive oxygen species disrupt ER homeostasis, triggering the unfolded protein response (UPR). As comprehensively reviewed by Ji et al.,^37^ ER stress and the UPR integrate metabolic, inflammatory, and proteostatic signals, driving a cascade that can amplify mitochondrial damage and propagate organ injury. This interaction establishes a vicious cycle where ER stress exacerbates mitochondrial ROS production, and mitochondrial dysfunction further impairs ER function, collectively contributing to the end-organ damage observed in diabetic panvascular disease.

In this context, the AMPK/SIRT1/PGC-1α signaling axis emerges as a central “metabolic hub” that integrates energy sensing, mitochondrial biogenesis, and epigenetic regulation. As comprehensively reviewed by Chen et al.,^38^ this axis directly couples the cellular energy status (indicated by the AMP/ATP ratio) to mitochondrial function and gene expression through AMPK-mediated phosphorylation and subsequent activation of the NAD^+^-dependent deacetylase SIRT1. PGC-1α acts as a downstream transcriptional coactivator of SIRT1, orchestrating mitochondrial biogenesis and the expression of antioxidant genes. Importantly, this pathway exhibits significant tissue-specific regulatory patterns, which directly influence cellular vulnerability across different vascular beds—for instance, the functional integrity of cardiomyocytes and neurons relies more heavily on this pathway compared to certain renal cell types.

### Epigenetic and post-translational modifications

Metabolic disturbances in diabetes leave long-lasting marks on the epigenome, a phenomenon known as “metabolic memory”. This explains why prior poor glycemic control confers a persistent risk of complications even after glucose normalization.^39^^,^^40^ DNA methylation changes are prominent. In the diabetic heart, hyperglycemia-induced downregulation of the protective antioxidant gene *JunD* is mediated by promoter hypermethylation, together with repressive histone modifications and miRNA-mediated translational inhibition.^1^ Conversely, pathological upregulation of the calcium regulator *SLN* in the diabetic heart post-MI is linked to promoter hypomethylation due to decreased DNA methyltransferase expression.^41^ In the kidney, high glucose induces DNA methyltransferase 1, leading to hypermethylation of the *Dao1* promoter and reduced D-amino acid oxidase expression, thereby contributing to retinal neuropathy.^42^

Non-coding RNAs, particularly miRNAs and lncRNAs, are key epigenetic regulators. Profiling of dysregulated miRNAs in the serum and retina of diabetic mice has identified several miRNAs that target key genes such as *VEGF*, *BDNF*, and *PPAR-α*, and these changes occur before overt retinopathy develops.^43^ In DCM, lncDACH1 is upregulated and promotes mitochondrial oxidative stress and apoptosis by binding to and promoting the degradation of the deacetylase SIRT3.^1^ In diabetic kidney disease, miR-1207-5p and other miRNAs are dysregulated.^44^ Exosomal miRNAs from various sources, including mesenchymal stem cells^45^ and urine,^46^ have shown promise as non-invasive biomarkers, while their potential as therapeutic agents is currently under preclinical investigation. For example, MSC-derived exosomes ameliorate diabetic peripheral neuropathy by delivering anti-inflammatory miRNAs that target the TLR4/NF-κB pathway.^45^ However, it must be acknowledged that exosome-based therapies for DPD remain at an early preclinical stage; to date, no completed phase I trial has specifically evaluated exosome therapy for diabetic heart disease, kidney disease, retinopathy, or peripheral neuropathy as a primary indication. The translational gap from rodent models to human DPD is substantial, and key hurdles (standardized isolation, dosing, tissue targeting, and long-term safety) remain unresolved. Thus, while exosomes hold promise as a delivery platform for miRNA-based interventions, their clinical readiness for DPD should not be overstated.

### Cross-organ regulatory nodes: Under-explored connections

Although the core metabolic pathways have been extensively studied within individual organs, the cross-organ nodes linking dysfunction across vascular territories remain insufficiently explored. Several emerging connections merit attention. The gut-vascular axis, involving microbial metabolites such as phenyl sulfate and TMAVA, can influence distant organ metabolism and may amplify DPD through bidirectional feedback. Organ-to-organ metabolic signaling, such as cardiokines from the diabetic heart or inflammatory mediators from the retina, may propagate injury across multiple beds. Systemic inflammatory and oxidative stress waves triggered by acute metabolic perturbations can synchronize damage responses in different vascular territories, potentially explaining the clinical co-occurrence of complications. Elucidating these cross-organ mechanisms could reveal novel therapeutic targets that interrupt the propagation of injury across vascular beds, offering a more comprehensive approach than targeting individual organs in isolation.

In recent years, the understanding of diabetic panvascular disease (DPD) has shifted from a solitary, organ-limited perspective toward a multi-organ interactive system network driven by a systemic circulatory hub. This cross-organ interaction network not only encompasses the classical cardio-renal axis, nephro-retinal axis, retina-brain axis, and neuro-renal axis but also integrates circulating metabolites, exosomes, and gut microbiome-derived signals, collectively forming a highly complex and dynamic pathogenic network.

At the macro-circulatory level, the gut-vascular axis extensively participates in regulating systemic inflammation, albuminuria, fatty acid oxidation (FAO), and cardiac hypertrophy through the release of gut-derived metabolites such as phenyl sulfate, TMAVA, short-chain fatty acids (SCFAs), bile acids, and LPS/LBP, thereby closely linking gut microbiota dysbiosis to multi-organ vascular lesions. Meanwhile, this network is profoundly regulated by the epigenetic mechanisms of “Metabolic Memory”: early hyperglycemia (Years 0–5), by inducing DNA methylation, histone modifications, and miRNA dysregulation, can sustain persistent cross-organ vulnerability for over a decade (Years 10+) even after subsequent glycemic control is achieved. This provides a key mechanistic fulcrum for explaining why patients with diabetes remain at high cardiovascular risk despite reaching glycemic targets.

At the micro-cellular communication level, exosomes, as emerging long-distance communication vehicles, demonstrate significant potential for transmitting pathological signals. Current evidence indicates that MSC-derived exosomes carry anti-inflammatory miRNAs, urinary exosomal CCL21 can serve as a novel biomarker, and cardiac-derived exosomes participate in the regulation of microvascular injury in distant organs such as the kidney and retina via the miR-133a signaling pathway. Notably, **sex differences** also play a non-negligible modulatory role in this network: estrogen, by regulating mitochondrial dynamics, predisposes females toward mitochondrial fusion (a protective mechanism), whereas males tend toward fission (accelerated cellular aging). This difference suggests that therapeutic strategies for DPD urgently require the incorporation of sex-specific therapy strategies.

In summary, these novel mechanistic discoveries that transcend organ boundaries and involve metabolic memory and the gut microbiome have significantly revolutionized our understanding of DPD pathophysiology. To thoroughly track this progress, we have systematically integrated recent original research and reviews published in authoritative international journals during this revision, replacing some outdated literature to reflect the latest frontiers in this field. Figure 3 summarizes the concept of metabolic memory, in which epigenetic marks drive persistent cross-organ vascular vulnerability.

**Figure 3:**
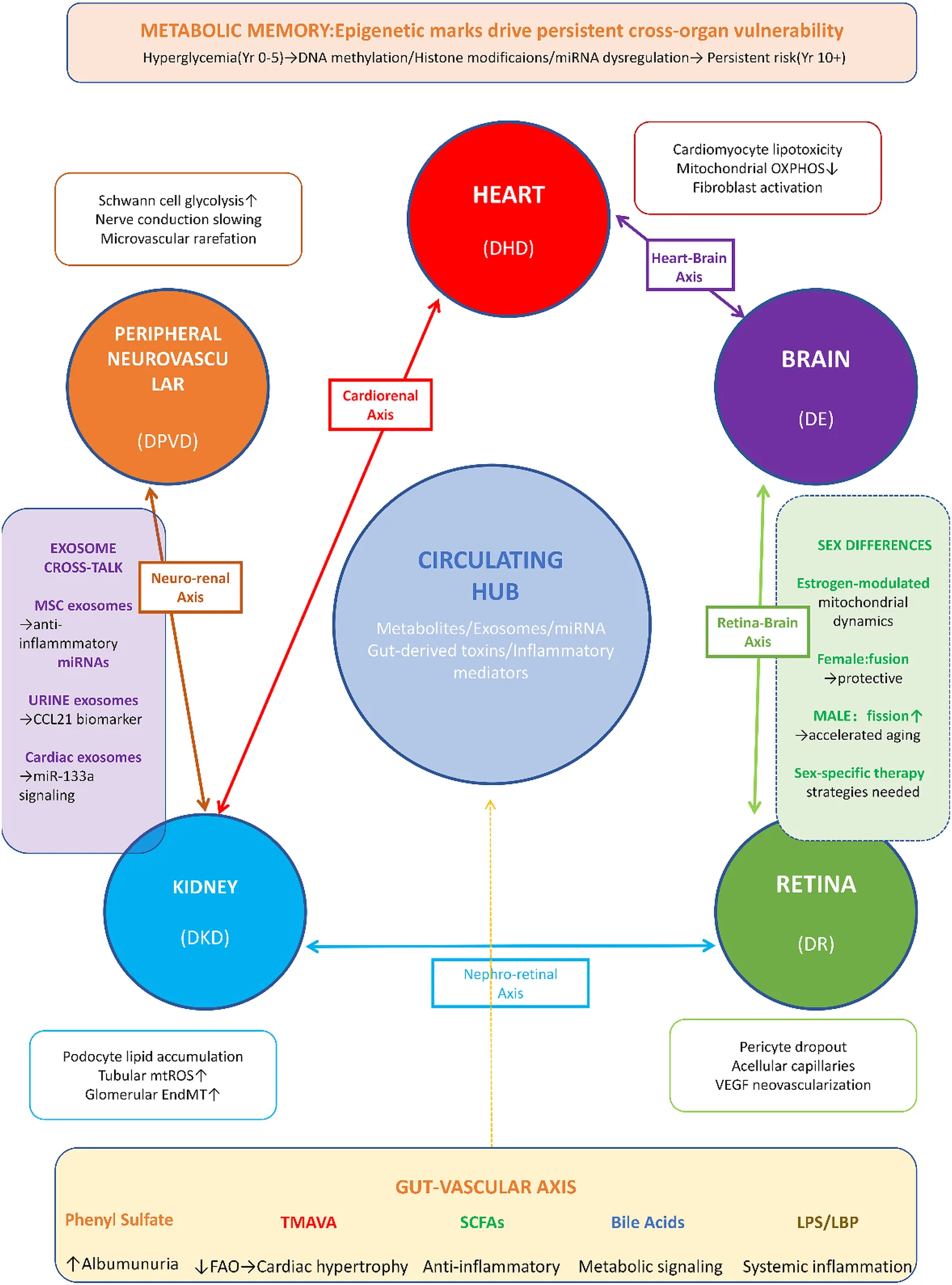
Multi-organ interactive network in diabetic panvascular disease (DPD) The systemic circulatory hub (metabolites, exosomes, miRNAs, gut-derived toxins, inflammatory mediators) drives cross-organ axes (cardio-renal, heart-brain, retina-brain, nephro-renal, and neuro-renal). Key mechanistic modules highlighted include: (1) epigenetic Metabolic Memory induced by early hyperglycemia; (2) Exosome Cross-talk mediating distant pathological signals; (3) Sex Differences in estrogen-modulated mitochondrial dynamics; and (4) the Gut-Vascular Axis linking gut microbiota dysbiosis to systemic inflammation and multi-organ lesions.

### Sex differences in vascular metabolic reprogramming

Sex differences represent an important yet under-appreciated dimension of metabolic reprogramming in DPD. A growing body of evidence indicates that estrogen, through its receptors (ERα, ERβ, and GPER), exerts female-specific protective effects on vascular metabolism, primarily via the regulation of mitochondrial function. Estrogen promotes mitochondrial biogenesis through the AMPK-PGC-1α pathway, enhances mitochondrial efficiency, reduces oxidative stress in the vasculature, and maintains mitochondrial network homeostasis by promoting fusion (via Opa1, Mfn1, Mfn2) while attenuating fission (via Drp1). In diabetic states, these estrogen-mediated protective mechanisms are progressively lost, particularly after menopause, which contributes to the accelerated cardiovascular risk observed in postmenopausal diabetic women. Conversely, male patients with diabetes exhibit distinct metabolic vulnerabilities, including greater mitochondrial dysfunction and contractile impairment in the absence of estrogen-mediated protection. The clinical significance of these sex differences is underscored by observations that diabetic women face a higher relative risk of coronary heart disease compared to diabetic men, yet this sex disparity diminishes after menopause, implicating estrogen deprivation as a key factor. Emerging therapeutic strategies, such as GPER agonists, have shown promise in reversing mitochondrial dysfunction in ovariectomized diabetic models, suggesting potential for targeting estrogen-related pathways in postmenopausal women with DPD. Collectively, these findings indicate that sex-specific metabolic regulation, particularly estrogen-mediated mitochondrial dynamics, represents a critical but under-explored node in DPD pathogenesis and therapy.

### Other emerging concepts: Single-cell flux and intervenable biomarkers

The development of single-cell technologies is providing unprecedented insight into the metabolic heterogeneity of cells within the vascular niche. For example, single-cell RNA sequencing of endothelial cells from patients with breast cancer identified a distinct capillary EC subtype (LIPEC) involved in lipid processing, whose abundance correlated with clinical benefit from metformin.^47^ This highlights the potential of single-cell approaches to uncover cell-type-specific metabolic programs and their responses to therapy. Similarly, circulating biomarkers that reflect specific aspects of metabolic reprogramming are being validated. In addition to those already mentioned, copeptin, a surrogate for arginine vasopressin, is emerging as an early biomarker for DKD.^48^ Urinary biomarkers of tubular injury, such as KIM-1^49^^,^^50^ and the EGF/MCP-1 ratio,^51^ offer prognostic value beyond albuminuria. The convergence of high-resolution “omics” technologies with functional metabolic studies promises to reveal a new generation of targets and biomarkers rooted in the metabolic reprogramming of specific vascular cell populations.

## Cell-type-specific metabolic reprogramming in DPD

The systemic metabolic dysregulation of diabetes manifests as distinct pathological processes in different vascular beds, driven by the unique metabolic requirements and vulnerabilities of the resident cell types. Here we provide a cross-organ comparative analysis that highlights how the same core metabolic abnormalities (glucose redirection, lipotoxicity, mitochondrial dysfunction) yield divergent pathological outcomes depending on the cell type and tissue context. Three cross-cutting themes emerge from this comparison. First, the energy dependency of the tissue determines its dominant metabolic vulnerability: the constantly contracting cardiomyocyte is exquisitely sensitive to substrate inflexibility and lipotoxicity, whereas the post-mitotic neuron is vulnerable to insulin signaling deficiency and barrier disruption, and the high-energy-demanding podocyte and proximal tubular cell are susceptible to lipid accumulation and mitochondrial oxidative stress. Second, the temporal dynamics of injury differ markedly across organs: microvascular changes in the retina and kidney may precede overt clinical disease by years, while cardiac dysfunction often manifests later but progresses more rapidly once established. Third, the relative contribution of each metabolic pathway varies substantially between vascular beds: the polyol pathway and PKC activation predominate in the retina, lipotoxicity and mitochondrial dysfunction are the major drivers in the heart, and the AGE-RAGE axis appears universally engaged but with organ-specific downstream effectors. These comparative insights, which are not evident from examining individual pathways or organs in isolation, underscore the importance of adopting a cross-organ perspective when targeting metabolic reprogramming in DPD.

Beyond these tissue-specific metabolic pathways, the reprogramming of innate immune cells, particularly macrophages, serves as a central amplifier of sterile inflammation across all vascular beds in DPD. Emerging evidence indicates that the cGAS-STING signaling pathway acts as a critical bridge linking mitochondrial dysfunction to immune activation. Under diabetic conditions, metabolically stressed cells release mitochondrial DNA (mtDNA) into the cytosol, which is sensed by cyclic GMP-AMP synthase (cGAS), leading to STING activation and subsequent production of type I interferons and pro-inflammatory cytokines via the TBK1-IRF3 and NF-κB axes. As systematically reviewed by Liu et al.^52^ this cGAS-STING axis critically regulates macrophage M1/M2 polarization dynamics, promoting a pro-inflammatory M1 phenotype that drives the secretion of IL-1β and TNF-α, thereby exacerbating the inflammatory cascade central to diabetic panvascular disease. Furthermore, the broader significance of this pathway in sterile inflammation and metabolic stress has been highlighted by Jiang et al.,^53^ demonstrating its role as a central regulator and a novel therapeutic target in systemic pathophysiology. These mechanistic insights underscore that targeting the cGAS-STING-macrophage axis may offer a novel strategy to simultaneously attenuate inflammatory cascades across the heart, kidney, and retina, providing a unifying immune-metabolic node for cross-organ intervention.

In parallel with this unifying immune-metabolic node, it is crucial to recognize that macrophages themselves are not a monomorphic population; rather, they exhibit a spectrum of activation states that can exert dual and often opposing effects in DPD. As systematically outlined by Shang et al.,^54^ macrophage polarization is more nuanced than a simple M1/M2 dichotomy. While M1 macrophages drive tissue destruction through the secretion of IL-1β, TNF-α, and ROS, M2-like macrophages can promote tissue repair, angiogenesis, and resolution of inflammation under appropriate conditions. However, in the diabetic microenvironment, the balance is heavily skewed toward the M1 phenotype, whereas the protective M2 functions are often impaired or overwhelmed. Thus, the “dual role” of macrophages—shifting from a protective reparative cell to a pathogenic driver—is not a fixed identity but a dynamic response to the metabolic milieu. This dynamic interplay highlights the need for therapeutic strategies that not only inhibit pathogenic M1 polarization but also restore or amplify the beneficial M2-like functions, rather than simply suppressing all macrophage activity.

In diabetic heart disease, the constant work of the cardiomyocyte makes it exquisitely sensitive to changes in energy substrates. Insulin resistance and hyperglycemia force the heart into a state of high FAO and relative glucose oxidation deficiency.^1^^,^^16^ This metabolic inflexibility, while initially compensatory, leads to lipotoxicity from accumulation of lipid intermediates, placing a burden on mitochondria and increasing ROS production.^1^ Mitochondrial dysfunction, characterized by impaired respiration and ATP synthesis, is a hallmark of the diabetic heart.^33^

The resultant oxidative stress and inflammation, driven by AGE RAGE and PKC signaling, promote the activation of a multi-modal regulated cell death (RCD) network in cardiomyocytes. As highlighted by Qi et al.,^36^ this network integrates apoptosis, necroptosis, ferroptosis, pyroptosis, and cuproptosis, whose distinct yet interconnected executioner pathways are synergistically activated by mitochondrial oxidative stress and lipotoxicity in the diabetic heart. This multi-modal RCD activation, alongside cardiomyocyte hypertrophy and fibroblast activation, culminates in myocardial fibrosis and diastolic dysfunction. Endothelial cells in the coronary microvasculature also suffer from impaired NO bioavailability due to eNOS uncoupling and oxidative stress, thereby compromising myocardial perfusion.^4^ From a cross-organ perspective, the heart stands out for its reliance on mitochondrial oxidative phosphorylation and its intolerance to metabolic inflexibility. Unlike the retina or kidney, where microvascular pathology predominates, the diabetic heart features both microvascular dysfunction and intrinsic cardiomyocyte metabolic reprogramming, making it susceptible to a dual hit of impaired substrate utilization and compromised perfusion.

Diabetic encephalopathy links metabolic dysfunction to neurodegeneration and cognitive decline. The brain’s high metabolic rate and dependence on glucose make it vulnerable to both hyperglycemia and hypoglycaemia.^42^ Impaired insulin signaling in the brain, termed “type 3 diabetes”, impairs neuronal survival and synaptic plasticity.^55^^,^^56^ Microvascular damage is a key feature. Hyperglycemia and AGEs disrupt the blood-brain barrier by reducing tight junction proteins and compromising pericyte coverage, leading to increased permeability.^57^^,^^58^ This BBB dysfunction is linked to cognitive deficits in animal models.^57^ Inflammatory mediators from activated microglia, driven by pathways such as TLR4/Akt/mTOR^59^ and TREM-2,^60^ contribute to neuronal injury. Furthermore, ER stress plays a central role in this neuroinflammatory cascade. As highlighted by Chen et al.^61^ in the context of neurodegeneration, ER stress engages in intensive crosstalk with neuroinflammation, where UPR activation in microglia and neurons amplifies pro-inflammatory cytokine release and exacerbates synaptic dysfunction. This ER stress-neuroinflammation axis provides a crucial mechanistic link between systemic metabolic reprogramming and progressive cognitive decline, suggesting that targeting ER stress pathways may offer a novel strategy for mitigating diabetic encephalopathy. Retinal microvascular parameters, such as reduced arteriolar fractal dimension, are independently associated with MACE in T2DM, suggesting that the retina may serve as a window into cerebral microvascular health.^62^ Notably, the brain shares with the retina a continuous microvascular endothelium characterized by tight junctions and a high degree of metabolic activity. However, unlike the retina, the brain is additionally threatened by impaired insulin signaling that directly compromises neuronal survival and synaptic function, introducing a neurodegenerative component that is not a primary feature of other diabetic complications.

In diabetic kidney disease, the metabolic insult targets the complex filtration unit. Podocytes, with their limited regenerative capacity, are particularly vulnerable. They accumulate lipids, a process driven by JAML-mediated SREBP1 activation and SIRT1 inhibition, leading to injury and proteinuria.^18^ Under diabetic conditions, activation of RANK in podocytes promotes NADPH oxidase expression (NOX4) via p65, exacerbating oxidative stress.^63^ The fenestrated glomerular endothelium is also damaged. Endothelial dysfunction is central, with markers such as endotrophin strongly predicting renal outcomes.^64^ EndMT contributes to fibrosis.^15^ Mesangial cells expand and produce excess matrix in response to hyperglycemia and TGF-β, driven by metabolic pathways including PKC and AGEs. Proximal tubular cells, responsible for high-energy reabsorptive work, undergo metabolic stress. Lipotoxicity from accumulated lipids,^17^ mitochondrial dysfunction from mtROS,^34^ and a hypoxic environment together lead to tubular injury and interstitial fibrosis. The kidney differs from the heart and brain in that its injury is driven by a combination of hemodynamic changes (glomerular hyperfiltration), metabolic dysregulation, and a unique anatomical feature, the fenestrated endothelium, which renders it particularly susceptible to filtration-related stress. Moreover, the coexistence of multiple metabolically active cell types (podocytes, mesangial cells, endothelial cells, tubular epithelial cells) within a single organ makes DKD a particularly informative model for understanding how distinct cell types respond to the same diabetic milieu through divergent metabolic programs.

Collectively, this cross-organ comparison reveals that while the core metabolic pathways are shared across vascular territories, their relative contributions, temporal dynamics, and downstream effectors are highly tissue-specific. These differences have direct therapeutic implications: a single metabolic modulator may exert organ-specific effects, and optimal DPD management will likely require tissue-targeted or context-dependent approaches.

## Therapeutic strategy map: From glycemic control to metabolic targeting

Based on the “pathway–organ–cell” framework described above, Table 1 summarizes the druggable targets relevant to DPD and their translational progress, covering marketed drugs (e.g., SGLT2 inhibitors, GLP-1 receptor agonists, metformin, RAAS inhibitors, anti-VEGF agents) as well as mechanism-oriented candidate strategies (e.g., inhibitors of AR, RAGE, PKC, NOX, NLRP3, JAK, mTOR, miRNA modulators). These targets correspond to key pathological nodes including glucotoxicity, lipotoxicity, oxidative stress, inflammation, hemodynamics, and epigenetics, suggesting that future therapeutic strategies need to move from simple glucose lowering toward multi-pathway, cross-organ metabolic precision interventions.

**Table 1:** Drug targets, level of evidence, and clinical translation progress in diabetic panvascular disease

Drug class/strategy	Primary molecular target	Priority disease coverage	Key mechanism	Translational tier	Clinical status	PMID(s)
**Tier I: Guideline-recommended therapies with established outcome data**						

SGLT-2 inhibitors	SGLT2	DKD, DHD, DE, DR, DPVD	Reduce renal glucose reabsorption, improve glycemic control, modulate mitochondrial OXPHOS, and confer cardiorenal protection	I	Approved (guideline-recommended)	Ma et al.^16^; Banks et al.^56^; Rom et al.^57^
GLP-1 receptor agonists	GLP-1R	DHD, DE, DPVD	Enhance insulin secretion, suppress glucagon, cross the BBB, and provide neuroprotection	I	Approved (guideline-recommended)	Rom et al.^58^
Metformin	AMPK/mTORC1	DHD, DE	Inhibits mitochondrial complex I, improves insulin resistance, and rewires cellular energy metabolism	I	Approved (guideline-recommended)	Chen et al.^61^
RAAS inhibitors (ACEI/ARB)	RAAS	DKD, DHD	Block angiotensin II signaling, lower intraglomerular pressure, reduce proteinuria, and provide cardiovascular protection	I	Approved (guideline-recommended)	Samaras et al.^65^; Zhang et al.^60^
Anti-VEGF agents	VEGF	DR	Inhibit pathological neovascularization and reduce vascular leakage	I	Approved (guideline-recommended)	Orkaby et al.^66^; Yu et al.^67^
PPAR-γ agonists (thiazolidinediones)	PPAR-γ	DHD, DE	Improve glucose metabolism, energy homeostasis, and inflammatory signaling	I	Approved	Zinman et al.^68^
DPP-4 inhibitors	DPP-4	DHD	Prolong endogenous GLP-1 activity and promote insulin secretion, with modest cardiovascular benefit	I	Approved	Ke et al.^63^; Chauhan et al.^64^
PDE III inhibitor (cilostazol)	Cilostazol	DE	Mitigates oxidative stress and improves cerebrovascular injury	I	Approved (for other indications)	Täubel et al.^69^

**Tier II: Agents with positive phase 2/3 trial results awaiting confirmatory outcome data**						

Nonsteroidal mineralocorticoid receptor antagonists	MR	DKD	Block mineralocorticoid receptor signaling and reduce cardiorenal events	II	Approved/phase III-supported	Zhang et al.^60^
VAP-1 inhibitor (ASP8232)	VAP-1	DKD	Reduces albuminuria through vascular and inflammatory modulation	II	Phase II	Holman et al.^70^
miR-132 inhibitor (CDR132L)	miR-132	DKD, DCM	Improves heart failure phenotypes and may modulate fibrotic remodeling in the heart and kidney	II	Phase II	Das et al.^71^

**Tier III: Experimental candidates requiring further clinical validation**						

Glucokinase activators	GK	DPD	Activate glucokinase to restore glucose homeostasis and potentially protect the vasculature	III	In development	Cui et al.^59^
Aldose reductase inhibitors	AKR1B1	DR, DKD, DPVD	Suppress the polyol pathway, reduce sorbitol accumulation and oxidative stress, and protect microvessels	III	In development	Mauer et al.^8^
RAGE inhibitors	RAGE	DCM, DKD, DR, DE	Block AGE-RAGE signaling, dampen oxidative stress and inflammation, and inhibit NF-κB activation	III	Research stage	Fitchett et al.^72^; Neal et al.^73^
PKC inhibitors	PKC	DR, DCM	Reduce polyol-pathway-associated oxidative stress, vascular permeability, and aberrant angiogenesis	III	In development	Suo et al.^11^; Wiviott et al.^74^
NADPH oxidase inhibitors	NOX	DPD	Decrease ROS generation and improve redox homeostasis	III	Research stage	Perkovic et al.^75^; Heerspink et al.^76^
eNOS modulators	eNOS	DE, DPVD	Improve vascular autoregulation and increase nitric oxide bioavailability	III	Research stage	Mathew et al.^23^; Palmer et al.^24^; Kidokoro et al.^77^
SIRT3 activators	SIRT3	DCM	Reduce mitochondrial oxidative stress and apoptosis	III	Research stage	Neal et al.^73^
NLRP3 inflammasome inhibitors	NLRP3	DCM	Suppress inflammasome-driven inflammation, fibrosis, and cardiac dysfunction	III	Research stage	Tashkandi et al.^31^; Zhang et al.^78^
PARP inhibitors	PARP	DPD	Improve DNA repair responses and alleviate hyperglycemia-induced vascular injury	III	Research stage	Marso et al.^79^
TBK1/IKKε inhibitors (amlexanox)	TBK1/IKKε	DPD	Improve metabolic dysfunction and lower HbA1c/fructosamine	III	Research stage	Husain et al.^80^
CD36 inhibitors	CD36	DPD	Reduce fatty acid uptake and foam-cell formation	III	Research stage	Fu et al.^18^; Liao et al.^19^
CXCR4 modulators	CXCR4	DPD	Regulate immune-cell recruitment to the vessel wall and may protect both macro- and microvasculature	III	Animal/early clinical	Shan et al.^81^
Glutamine metabolism modulators	GLS/GS	DPD	Regulate endothelial proliferation, angiogenesis, and plaque biology	III	Research stage	Kass et al.^82^; Vaccaro et al.^83^
Asparagine synthetase modulators	ASNS	DPD	Asparagine and α-ketoglutarate supplementation may reverse endothelial injury	III	Research stage	Erdmann et al.^84^
MCP-1 inhibitors	MCP-1	DKD	Reduce inflammatory cell infiltration and slow renal fibrosis	III	Research stage	Zhu et al.^85^; Klein et al.^86^
miR-125a-5p mimic	miR-125a-5p	DR	Markedly attenuates vascular leakage	III	Research stage	Giblin et al.^87^
Serine palmitoyltransferase inhibitors	SPT	DPVD	Correct sphingolipid dysmetabolism and reduce neurotoxicity	III	Research stage	Qi et al.^36^
NMDA receptor modulators	NMDA	DE	Modulate neurogenesis and synaptic plasticity and may improve learning and memory	III	In development	Tuttle et al.^88^; Heerspink et al.^89^

DKD, diabetic kidney disease; DHD, diabetic heart disease; DE, diabetic encephalopathy; DR, diabetic retinopathy; DPVD, diabetic peripheral vascular disease; DPD, diabetic panvascular disease; DCM, diabetic cardiomyopathy; BBB, blood-brain barrier; OXPHOS, oxidative phosphorylation; ROS, reactive oxygen species; AGE, advanced glycation end-product; RAGE, receptor for AGE; NF-κB, nuclear factor kappa-B; PKC, protein kinase C; eNOS, endothelial nitric oxide synthase; NOX, NADPH oxidase; NLRP3, NOD-like receptor family pyrin domain containing 3; PARP, poly(ADP-ribose) polymerase; TBK1, TANK-binding kinase 1; IKKε, inhibitor of nuclear factor kappa-B kinase subunit epsilon; CD36, cluster of differentiation 36; CXCR4, C-X-C chemokine receptor type 4; GLS, glutaminase; GS, glutamine synthetase; ASNS, asparagine synthetase; MCP-1, monocyte chemoattractant protein-1; NMDA, N-methyl-D-aspartate; SPT, serine palmitoyltransferase.

### Evidence-based therapies: Pleiotropic protection beyond glycemic control

Currently, the cornerstone of DPD treatment has expanded from simple glycemic control to drugs with proven vascular protective effects. Metformin, as a first-line agent, has its cardiovascular benefits supported by long-term studies. The 10-year follow-up of the UKPDS trial showed that in newly diagnosed overweight patients with T2DM, intensive metformin therapy not only reduced microvascular complications but also significantly reduced the risk of myocardial infarction and all-cause mortality, reflecting an early “legacy effect”.^70^ Moreover, in patients with diabetes, metformin use is associated with a lower risk of dementia, particularly in younger (<75 years) patients.^65^^,^^66^ This neuroprotective effect may be related to its ability to improve endothelial progenitor cell function and accelerate wound healing.^67^

The advent of SGLT2 inhibitors and GLP-1 receptor agonists has revolutionized the DPD treatment landscape. SGLT2 inhibitors (e.g., empagliflozin, canagliflozin, dapagliflozin) have shown significant cardiorenal protective effects in multiple large cardiovascular outcome trials. The EMPA-REG OUTCOME trial first demonstrated that in patients with T2DM at high cardiovascular risk, empagliflozin not only reduced the risk of major adverse cardiovascular events but also significantly reduced cardiovascular death and hospitalization for heart failure.^68^ This benefit extended to patients with or without a history of heart failure.^72^ The CANVAS program also confirmed the efficacy of canagliflozin in reducing cardiovascular event risk, although an increased risk of amputation was noted.^73^ The DECLARE-TIMI 58 trial showed that dapagliflozin reduced the composite endpoint of cardiovascular death or hospitalization for heart failure, primarily driven by a reduction in heart failure hospitalization.^74^ Regarding kidney protection, the CREDENCE trial first demonstrated that in patients with diabetic kidney disease, canagliflozin significantly reduced the risk of end-stage renal disease, doubling of serum creatinine, or renal death.^75^ The DAPA-CKD trial extended the benefit to patients with chronic kidney disease with or without type 2 diabetes.^76^ The mechanisms of SGLT2 inhibitors are complex and include reduction of intraglomerular pressure via tubuloglomerular feedback,^77^ weight loss, improvement in energy metabolism, and potential anti-inflammatory and anti-fibrotic effects.^78^

GLP-1 receptor agonists also exhibit robust cardiovascular and renal protective effects. The LEADER trial showed that liraglutide significantly reduced the risk of cardiovascular death, myocardial infarction, or stroke in patients with high-risk T2DM.^79^ The REWIND trial demonstrated that dulaglutide reduced the risk of major cardiovascular events and slowed the progression of renal outcomes in a broad T2DM population (including a majority without prior cardiovascular disease).^79^ Oral semaglutide confirmed its cardiovascular safety in the PIONEER 6 trial.^80^ The SUSTAIN-6 trial showed that semaglutide significantly reduced cardiovascular event risk.^79^ The benefits of GLP-1 RAs are thought to be partly due to their anti-inflammatory effects, improvement in endothelial function, and stabilization of atherosclerotic plaques.^81^

### Mechanism-driven future therapies: Targeting metabolic hubs

Based on a deep understanding of metabolic reprogramming mechanisms, a series of new therapies targeting specific pathways are under active development. Table 1 summarises the major drug targets that are either already on the market or in clinical development, along with their mechanisms of action.

Targeting glucotoxicity: Aldose reductase inhibitors (e.g., epalrestat) have been used in some countries to treat diabetic neuropathy by inhibiting the overactivity of the polyol pathway.^5^ The AGE-RAGE axis is also a popular target. For example, RAGE antagonists or cross-link breakers (e.g., ALT-711) have shown potential to improve arterial compliance in early studies.^82^ In addition, drugs such as cilostazol have been shown to improve high-glucose-induced vascular smooth muscle dysfunction by inhibiting the RAGE signaling pathway.^1^

Targeting lipotoxicity and energy metabolism: PPAR agonists such as pioglitazone act by improving insulin sensitivity, but attention must be paid to potential risks of heart failure and bladder cancer.^83^^,^^84^ Glucokinase activators (e.g., dorzagliatin) aim to restore the rhythmicity of glucose sensing and utilization and have shown good glucose-lowering efficacy and safety in clinical trials.^85^^,^^86^

Targeting mitochondria and oxidative stress: Mitochondria-targeted antioxidants (e.g., MitoQ) have shown potential to alleviate DKD and diabetic cardiomyopathy in preclinical models.^34^ Inhibiting PKC-β (e.g., ruboxistaurin) can improve endothelial function, promote angiogenesis, and accelerate wound healing in diabetic mice.^71^

Anti-inflammatory strategies: Interventions targeting inflammatory pathways are being explored. For example, IL-1β antagonists^87^ and JAK inhibitors (e.g., baricitinib) reduced proteinuria in a phase 2 study of patients with DKD.^88^ Endothelin A receptor antagonists (e.g., atrasentan) reduced the risk of major renal events in patients with diabetic kidney disease in the SONAR trial, but fluid retention side effects must be monitored.^89^

Epigenetic and non-coding RNA-based strategies: miRNA-based therapies (e.g., the miR-132 antagonist CDR132L) have shown potential to improve cardiac function in a phase 1b study of heart failure.^69^ Exosome therapy, particularly using mesenchymal stem cell-derived exosomes, has successfully ameliorated diabetic peripheral neuropathy^45^ and promoted wound healing^90^ in preclinical models by delivering anti-inflammatory miRNAs.

### Translational gaps: Lessons from failed clinical trials

Despite robust preclinical evidence for many mechanism-based targets, translation to clinical success has been challenging. NLRP3 inflammasome inhibitors consistently attenuate cardiac and renal injury in rodent DPD models, yet human trials have failed. This discrepancy may reflect species differences in inflammasome activation thresholds (murine macrophages show lower thresholds than human macrophages), suboptimal dosing or timing in clinical studies, and the multifactorial nature of human DPD, where NLRP3 inhibition alone cannot modify decades-long disease trajectories. Aldose reductase inhibitors (ARIs) such as sorbinil showed pronounced efficacy in animal models of retinopathy and nephropathy, but failed in large human trials. Possible reasons include suboptimal tissue penetration of first-generation ARIs, compensatory activation of parallel pathways (AGE-RAGE, PKC) when the polyol pathway is blocked, and intervention after irreversible structural damage is established. RAGE antagonists also face translational hurdles; despite efficacy in preclinical diabetes models, no agent has completed pivotal phase 3 trials. Explanations include the heterogeneous nature of AGEs, redundant receptor-ligand interactions, and compensatory signaling that bypasses RAGE. Collectively, these failures highlight the need for careful species-specific model selection, evaluation of target engagement and pathway redundancy, appropriate patient stratification, and combination therapy that targets multiple complementary pathways in complex DPD.

### A four-tier stratified targeted therapy strategy and clinical translation framework based on metabolic reprogramming

To systematically address the core challenges of target prioritization, patient subgroup stratification, and combination therapy design, we propose a novel “Metabolic Reprogramming-Targeted Therapy Four-Tier Stratified Roadmap” for diabetic panvascular disease, integrating the newly constructed. This framework systematically categorizes interventions currently at different developmental stages into four tiers: Tier 1, represented by SGLT2 inhibitors, GLP-1 receptor agonists, metformin, and RAAS blockers, exerts cross-organ, multi-target first-line foundational metabolic protective effects; Tier 2 focuses on approved organ-specific therapies, including anti-VEGF agents (for retinopathy), MRAs (for diabetic kidney disease), as well as anti-inflammatory and antiplatelet drugs, targeting specific microvascular beds for precision intervention; Tier 3 encompasses mechanism-targeted drugs currently in clinical development (e.g., anti-fibrosis/endothelial protective agents, anti-glycotoxicity pathway inhibitors) aiming to overcome the limitations of traditional targets; and Tier 4 covers emerging preclinical targets, such as PARP inhibitors, cGAS-STING, CD36 inhibitors, and exosome therapy, representing the frontiers of exploration.

Based on this stratified framework, we further propose specific strategies: (1) For target prioritization, a rigorous assessment should be conducted based on the multi-organ benefit evidence and clinical translational risks of each candidate target (notably the “Translation Gaps” warnings highlighted at the top of Figure 4, such as the limited translation of AR inhibitors, Phase III failures of RAGE antagonists, and clinical setbacks for NLRP3 inhibitors), to prioritize safe and efficacious cross-organ targets; (2) For patient subgroup stratification, it is recommended to integrate multi-omics-guided precision phenotyping and single-cell metabolic flux technologies to identify the heterogeneity of metabolic reprogramming across different organs (heart, kidney, retina, and peripheral tissues), thereby matching patients to the optimal tier or drug combination; and (3) For combination therapy design, we advocate a “foundational overlay” principle. This entails strategically adding Tier 2 targeted drugs in a progressive manner based on the severity of organ involvement, while maintaining systemic metabolic stability with Tier 1 agents. Simultaneously, suitable patients can be screened for inclusion in Tier 3 and Tier 4 clinical trials, enabling a transition from single-pathway inhibition to multi-pathway synergistic intervention.

**Figure 4:**
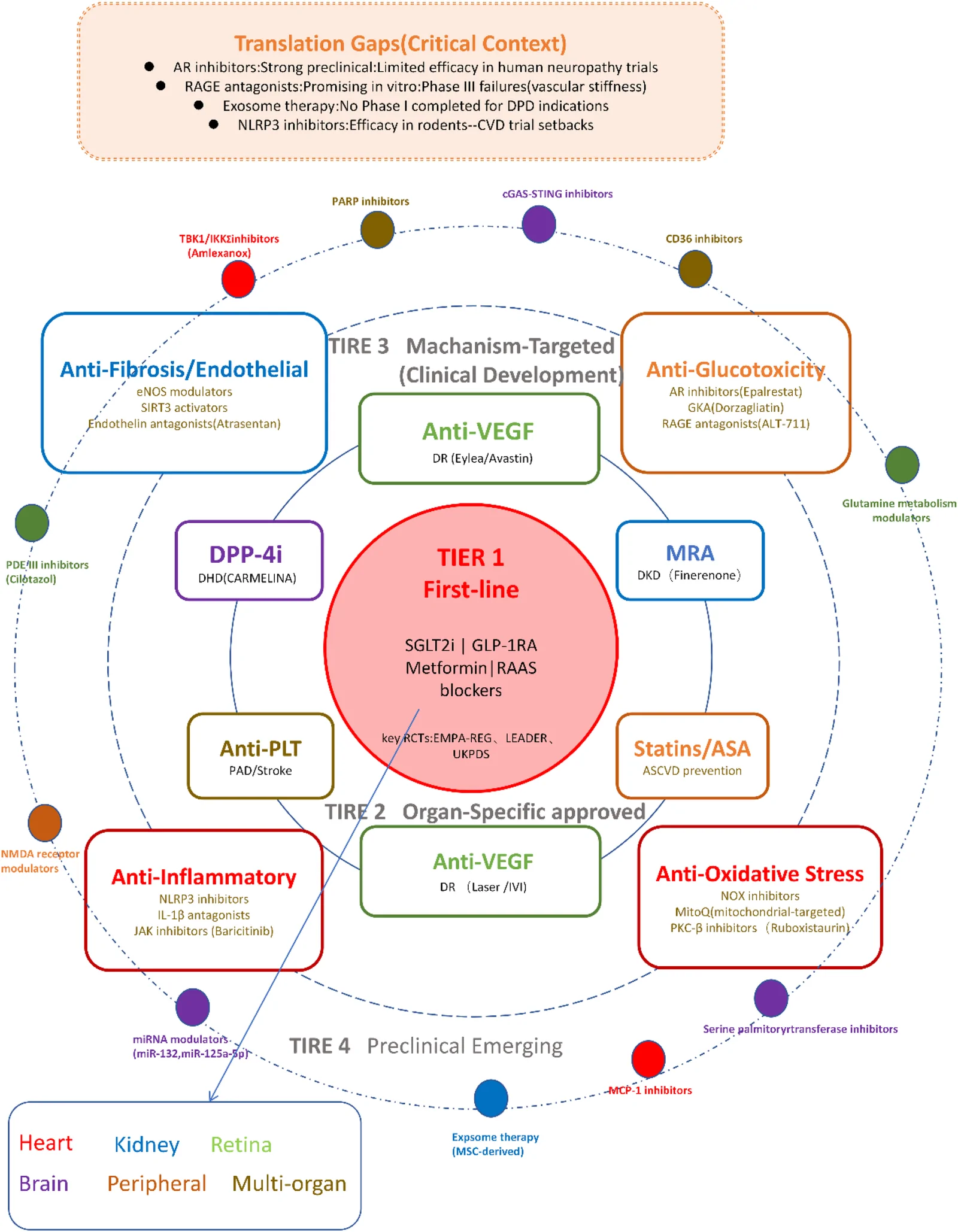
Four-tier stratified targeted therapy roadmap for DPD Interventions are systematically categorized into four translational tiers: Tier 1 (first-line foundational multi-organ metabolic protection, e.g., SGLT2i, GLP-1RAs, metformin, RAAS blockers); Tier 2 (organ-specific approved therapies); Tier 3 (mechanism-targeted agents in clinical development); and Tier 4 (preclinical emerging targets). The outer ring denotes organ-specific targeting for the heart, kidney, retina, brain, and peripheral vasculature. The top “Translation Gaps” box highlights the critical clinical failures of many mechanism-targeted candidates despite robust preclinical efficacy.

Finally, we emphasize that future efforts should focus on multi-omics precision phenotyping, cross-organ causal inference, single-cell metabolic flux technologies, and adaptive clinical trial designs. By integrating the four-tier stratified framework and translational gap analysis provided in Figure 4, this review not only reshapes the understanding of the disease from a metabolic perspective but also effectively fills the gap left by previous “targeted therapy roadmaps” that lacked a systematic evaluation system, thereby offering a concrete operational guide for the precision intervention and clinical translation of diabetic panvascular disease.

## Unmet needs and future perspectives

Despite tremendous progress in understanding DPD, many unmet needs remain. First, the development of precision phenotyping and biomarkers is critical. Currently used clinical markers (e.g., HbA1c) are insufficient to fully predict individual responses to therapy or the risk of complications. We need to integrate multi-omics data (genomics, metabolomics, proteomics) to identify biomarkers that reflect specific metabolic pathway disturbances, thereby guiding personalized treatment. For example, although the urine proteomics classifier CKD273 score can predict the risk of developing microalbuminuria, spironolactone intervention guided by this score failed to achieve a positive result in the PRIORITY trial,^91^ indicating that more precise targets and intervention timing are still needed.

Second, causal inference of cross-organ networks is a key focus for future research. DPD is a complex network disease involving multiple organs. There are close epidemiological and mechanistic links between retinopathy and cerebrovascular disease,^92^ and between nephropathy and cardiovascular events. Advanced causal inference methods and systems biology models are needed to elucidate the causal relationships and synergistic effects among complications in different organs and to identify the key hubs that drive the deterioration of the entire network.

Third, the application of new technologies will deepen DPD research. Spatial transcriptomics and single-cell metabolic flux technologies can resolve the metabolic status and intercellular interactions of different cell types *in situ*, revealing early metabolic heterogeneity in lesions. For example, single-cell sequencing has already identified a specific endothelial cell subtype involved in lipid processing.^47^ These technologies will help us map the cellular landscape of DPD more precisely.

Finally, clinical trial design needs to be innovative. Real-world data and adaptive trial designs can more efficiently evaluate the long-term efficacy and safety of existing drugs (e.g., metformin, SGLT2 inhibitors, GLP-1 RAs) in different DPD subtypes and explore the potential of combination therapy. At the same time, therapies targeting completely new mechanisms, such as those targeting mitochondria,^93^ non-coding RNAs,^94^ and gut microbiota metabolites (e.g., phenyl sulfate^95^ or TMAVA^96^), need to be rapidly translated from preclinical studies to early clinical trials. In conclusion, the treatment of DPD stands at the dawn of a new era. Moving from a “glucose-centric” to a “vascular protection-centric, metabolic disorder-improving” paradigm, we are gradually translating the profound mechanistic insights into metabolic reprogramming into precise and effective therapeutic strategies that can bring greater clinical benefits to patients with DPD.

## Ethics and consent to participate

Not applicable.

## Consent for publication

Not applicable.

## Data and code availability

No new data were generated in the preparation of this narrative review. All information and supporting evidence presented herein were derived from previously published studies, as cited in the reference list.

## Acknowledgments

S.L. provided overall direction and guidance throughout the development of this manuscript. Y.B. and Y.B. performed the literature collection and interpretation and served as the primary contributors to the writing, editing, and revision of the manuscript. All authors reviewed and approved the final version of the article.

Funding: This work was supported by the Shanxi Provincial Special Project for Transformation of Scientific and Technological Achievements (202304021301066) and the Shanxi Provincial Key Research and Development Program Project (202402130501006).

## Declaration of interests

The authors declare that they have no competing interests.
